# Overcoming COVID-19 in China despite shortcomings of the public health system: what can we learn?

**DOI:** 10.1186/s13561-021-00319-x

**Published:** 2021-07-06

**Authors:** Mei Mei Wang, Steffen Fleßa

**Affiliations:** University of Greifswald, Greifswald, Germany

**Keywords:** COVID-19, Health care system, System dynamics model, Health codes, Contact-tracking technology

## Abstract

**Background and objective:**

The COVID-19 pandemic started in Wuhan, China, in December 2019. Although there are some doubts about the reporting of cases and deaths in China, it seems that this country was able to control the epidemic more effectively than many other countries. In this paper, we would like to analyze the measures taken in China and compare them with other countries in order to find out what they can learn from China.

**Methods:**

We develop a system dynamics model of the COVID-19 pandemic in Wuhan. Based on a number of simulations we analyze the impact of changing parameters, such as contact rates, on the development of a second wave.

**Results:**

Although China’s health care system seems to be poorly financed and inefficient, the epidemic was brought under control in a comparably short period of time and no second wave was experienced in Wuhan until today. The measures to contain the epidemic do not differ from what was implemented in other countries, but China applied them very early and rigorously. For instance, the consequent implementation of health codes and contact-tracking technology contributed to contain the disease and effectively prevented the second and third waves.

**Conclusions:**

China’s success in fighting COVID-19 is based on a very strict implementation of a set of measures, including digital management. While other countries discuss relaxing the lock-down at a rate of 50 per 100,000 inhabitants, China started local lock-downs at a rate of 3 per 100,000. We call for a public debate whether this policy would be feasible for more liberal countries as well.

## Introduction

In December 2019, a Pneumonia of unknown cause broke out in Wuhan, Hubei Province, China. The number of cases and deaths rose exponentially with tremendous challenges to the health care system and the society [1]. On January 7, 2020, the China Centers for Disease Control and Prevention (CDC) detected a new human coronavirus and sequenced the whole genome of the virus [2, 3], which was subsequently identified as the pathogen of the disease [4, 5]. On January 12, 2020, the World Health Organization (WHO) officially named the virus “Novel Coronavirus 2019” (2019-nCoV), and on February 11, 2020 the International Committee on The Classification of Viruses (ICTV) named the virus as SARS-CoV-2. The disease was subsequently named Corona Virus Disease 2019 (COVID-19).

As SARS-Cov-2 is highly infectious [6, 7], the new disease spread rapidly to other countries and continents and less than 3 months after the first reported cases in Wuhan WHO officially declared a COVID-19 pandemic [8, 9] (11.03.2020). However, while many countries and in particular Europe, North and South America are suffering from very strong waves of the disease with millions of cases and victims, China seems to have won the fight against the disease [10]. While one might challenge the quality and transparency of public health information from China, it is a matter of fact that China has comparably few cases of COVID-19. Outbreaks as we experience them in the United States of America (USA), Spain, Germany or France in particular in winter 2020/21 could not be hidden from WHO and the rest of the world.

Consequently, we have to ask for the reasons of this success. At a first glance, there might be three causes. Firstly, some external factors such as climate, genetics or culture might constitute a natural barrier against the diffusion of the disease. Secondly, a brilliant public health system with high resources might be capable to reduce the spread of the disease. Thirdly, specific interventions against the disease might have managed to control the outbreak in the country which are not consistently implemented in the most severely hit countries.

In this paper, we will analyze the relevance of these three factors. Consequently, the next section discusses the outbreak of COVID-19 in Wuhan in order to investigate external factors influencing the spread of the disease as well as the instruments applied in the region. As methods and results, we will present a simulation model of the diffusion of COVID-19 in Wuhan. Afterwards we will discuss the public health care system of China and its ability to produce results, which are more likely to control the disease than in Europe or the USA. We will also compare the instruments applied in China with other countries in order to determine the underlying causes of this success story.

## COVID-19 in Wuhan

Wuhan is the name of a city with about 8 million and of the respective metropolitan region of about 11 million inhabitants within the Hubei region of China. With a gross national product of about 23,000 US$ p.c. p.a., Wuhan is comparable wealthy. Wuhan is around 30^o^ North, such as Cairo, Jacksonville, New Orleans and Houston.

The origin and the first cases of COVID-19 in Wuhan are under dispute, but the disease became a medical issue in December 2019 [11]. China’s response to the epidemic can be divided into four stages according to the dynamic process of the epidemic, i.e., December 2019 to 19.01.2020, 20.01.-20.02.2020, 21.02.-28.04.2020 and 29.04.2020 until today.

### Phase I

Between December 2019 and 19th of January 2020, the number of patients grew exponentially, but it was too small that officials did not recognize it as a public health threat. When WHO was informed about the new disease of unknown etiology on 31st of December 2019, 27 patients had been diagnosed with viral pneumonia of unknown cause. On the 19th of January, already 198 patients had been recorded which is an increase of 11% per day (geometric mean).

In this phase, most official activities were confidential and not transparent, but the National Health Commission of China started with the pathogen detection and epidemiological investigation, issued technical guidelines to Wuhan, and notified other regions. However, it seems that Wuhan and other parts of China did not implement any significant epidemic prevention actions except for case detection and disease surveillance (Table 1). For instance, a community in Wuhan held the annual “10,000 Family Banquet” as scheduled on 18th of January.

**Table 1:** Measures taken in Wuhan in Phase I [12–17]

Day	Measures
Dec. 27	Cluster of Cases of pneumonia of unknown origin first reported to China CDC
Dec. 29	Pneumonia cases linked to the Huanan Seafood Wholesale Market
Dec. 30	Case-finding activated
Dec. 31	Outbreak announced by Wuhan Health Commission (WHC); National Health Commission (NHC) and China CDC involved in investigation and response
Jan. 01, 2020	Huanan Seafood Wholesale Market closed
Jan. 02	China CDC carried out pathogen identification
Jan. 03	Emergency monitoring, case investigation, close contact management, and market investigation initiated, technical protocols for Wuhan released; NHC notified WHO and relevant countries and regions; gene sequencing completed by China CDC
Jan. 06	China CDC Level 2 emergency response activated
Jan. 07	Coronavirus isolated and named 2019-nCOV
Jan. 08	A novel coronavirus was officially announced as the causative pathogen of the outbreak by China CDC
Jan. 09	The second group of experts from the NHC went to Wuhan to carry out epidemic prevention work
Jan. 10	China CDC publicly shared the gene sequence of the novel coronavirus; completed (Polymerase Chain Reaction) PCR diagnostic reagent development and testing; China begins its annual Spring Festival travel rush
Jan. 11	PCR diagnostic reagents provided to Wuhan; First fatal case reported
Jan. 14	The airport, railway station and wharf of Wuhan city carry out temperature check for departing passengers
Jan. 15	China CDC emergency response level upgraded to Level 1 (the highest level); national technical protocols for 2019-nCoV released by NHC
Jan. 16	Strict exit screening measures activated in Wuhan, people with body temperature 37.3 °C were restricted from leaving
Jan. 18	The third group of experts from the NHC went to Wuhan to carry out epidemic prevention work

### Phase II

Phase II was the phase of high incidence from 20th of January to 20th of February 2020. The emergence of COVID-19 coincided with the world’s largest annual human migration, the Chinese Spring Festival travel season. Under normal circumstances, China could have expected some 3 billion trips in China during the 40-day holiday period from 15 days before the Spring Festival to 25 days after the Spring Festival. For this “spring transit”, the Chinese language has even a special word called “Chunyun” where almost the entire population moves back to their place of origin. As the largest transportation hub in central China, Wuhan would have expected to transfer at least 15 million passengers during the Spring Festival holiday. According to Wuhan Transport Bureau, Wuhan counted a total of 15,223,900 passengers leaving Wuhan and 14,662,200 passengers arriving in Wuhan during the 2018 Spring Festival with a total of 271,339,500 travel activities during this period in Wuhan. There is no reason to assume that the situation would have been different in 2020 without COVID-19 and the Spring Festival 2020 could have become the super-spreader event for the entire country. In fact, it was reported that about 5 million people had already left Wuhan before the city was locked down on January 23, 2020 [18].

**Fig. 1:**
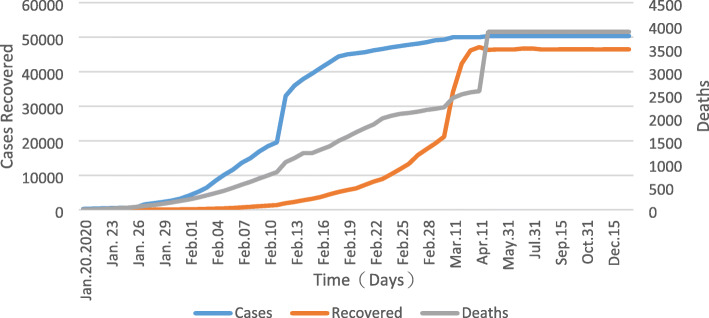
Confirmed and dead COVID-19 patients in Wuhan in Phase II-IV. Source: [28]

After it had been detected that the new disease was contagious [19, 20], the Government of China took emergency measures to reduce the risk of spreading the disease all over China, such as cancelling the Spring Festival, postponing work and school opening, restricting travel, closing entertainment venues and banning public gatherings (Table 2) [27]. However, during this period the number of cases and deaths strongly increased reaching a peak of 32,994 cases on 12th of February (Fig. 1). However, during this period the statistical detection of cases changed so that the jump from 11th to 12th of February is mainly due to different reporting than to a real increase of cases.

**Table 2:** Measures taken by China in Phase II [12, 15, 16, 21–26]

Day	Measures
Jan. 20	Novel Coronavirus Infected Pneumonia (NCIP) categorized as a Category B infectious disease and managed under Category A infectious diseases; Infection in health-care workers caring for 2019-nCoV patients; Close contacts of COVID-19 are monitored household in residential areas on a daily basis
Jan. 21	The central government and local governments at all levels bear the cost of hospital care for COVID-19 patients; Ministry of Transport launches Level 2 emergency Reagent probes and primers shared with the public by China CDC
Jan. 22	Hubei province launched a level II emergency response to a public health emergency; Wuhan residents must wear face masks in public places
Jan. 23	Wuhan city travel ban; first 3 provinces begin Level 1 response; Wuhan begins to seal off the city; Wuhan city traffic suspension; The airport and railway station are temporarily closed; Wuhan bans travel All public events have been cancelled
Jan. 24	Hubei province and other 14 provinces begin Level 1 response; Since that day, 346 national medical teams, 42,600 medical personnel and 965 public health workers have supported Wuhan and Hubei province
Jan. 25	13 provinces begin Level 1 response; Vehicles are prohibited in the central city of Wuhan; All kinds of emergency relief materials were dispatched to Wuhan
Jan. 26	China State Council approves an extension of the Spring Festival holidays; University, primary and secondary schools, kindergartens postponed the start of school
Jan. 27	Ministry of Education postpones start of the spring semester in 2020
Jan. 28	Ministry of Transport refunds all public rail, road, and water travel tickets
Jan. 29	Last province begins Level 1 response
Jan. 30	14,000 health checkpoints set up at bus and boat terminals, service centers, and toll gates nationwide
Feb. 02	Wuhan implements centralized treatment for confirmed patients, suspected patients, febrile patients and close contacts of confirmed patients, Conduct the most detailed screening
Feb. 03	Wuhan strives to build makeshift hospitals; Travel permits to Hong Kong and Macau suspended
Feb. 04	Huoshenshan hospital with 1000 beds was put into operation
Feb. 05	The makeshift hospital was opened for the first time to treat patients with mild COVID-19
Feb. 08	Leishenshan hospital with 1600 beds was put into operation
Feb. 10	19 provinces supported 16 cities, prefectures and county-level cities except Wuhan, Hubei province
Feb. 11	Residential districts in Hubei province put under closed management; Wuhan has reported 1102 confirmed cases among medical staff; Beijing, Tianjin, Shanghai and Chongqing have successively declared closed management of residential areas Health Code Model launched
Feb. 15	Seven diagnostic test reagents have been approved for market; some drug screening and treatment programs have made progress
Feb. 16	In Wuhan city, 11 makeshift hospitals have been put into operation in Wuhan city to meet the requirement of receiving as much as possible
Feb. 17	The Chinese government has deployed targeted prevention and control measures at different levels in different regions and departments to restore production and life order in an orderly manner
Feb. 18	The number of new cured and discharged cases in China exceed the number of new confirmed cases, and the number of confirmed cases begin to decline
Feb. 19	The number of new cured and discharged cases in Wuhan exceed the number of new confirmed cases for the first time
Feb. 20	The largest makeshift hospital in Wuhan was officially put into use, providing 3690 beds for patients with mild COVID-19

A crucial component of the intervention portfolio became the “Health Code Model”. On February 11, 2020, the Health Code model was first launched in Hangzhou, Zhejiang Province, China [29], and has been implemented in other provinces and cities in China since then. The smartphone-based application (app) registers the visited locations and all encounters and transfers it to a national database (whereby – from a western perspective – little value is placed on data protection rights). It gives the users an individual “health code” according to the traffic light scheme. The color green means that users can move around freely. Those who receive the yellow warning signal have to stay at home for 7 days, and red means a two-week quarantine [30]. These measures enable a relatively “normal” life, but they interfere with the privacy of citizens.

### Phase III

The third phase concentrated on stabilization of the epidemic (21 February - 28 April). As shown in Fig. 1 the incidence remained at a very low level since mid-March, and since April 1, there were no new infections. Consequently, the number of deaths also strongly declined. However, a new number of 1290 deaths was added in Wuhan on April 17, which was mainly a recalculation of the number of unreported deaths in the early stage of the epidemic, rather than the current number of deaths [31]. On April 26, all COVID-19 hospitalized cases in Wuhan had died or were discharged from the hospital. The epidemic situation was generally stable; the Chinese government began to coordinate the epidemic prevention and control with economic and social development, and to resume work and production.

### Phase IV

The interventions against COVID-19 in Wuhan are still in the fourth phase concentrating on early detection of cases and in particular efforts of epidemic prevention and control. However, Wuhan is not seen an exceptionality any longer but the countrywide measures to control the epidemic in the country are applied in Hubei Province as well. At this stage, China mainly focuses on “preventing import from outside and preventing rebound from inside” [22], so as to comprehensively promote the resumption of work, production and school, and restore the normal economic and social order. On April 30, the level of emergency response to public health emergencies in the Beijing-Tianjin-Hebei region was adjusted from level 1 to level 2, on June 13; the adjustment was lowered to level 3. On May 2, the emergency response level of public health emergencies in Hubei province was adjusted from level 1 to level 2. From May 14 to June 1, nearly 9.9 million nucleic acid tests were conducted in Wuhan, and no confirmed cases were found.

Meanwhile, according to the severity of the epidemic, the Chinese government splits up their counties (cities, districts) into low-risk areas, medium-risk areas and high-risk areas. High risk or medium risk areas can be very small units, such as a community, a street and a housing estate. The number is updated every day. As of February 17, China has dropped to 1 high-risk area and 5 medium risk areas.

As shown in Table 3, the specific prevention and control measures are adjusted according to the situation of provinces, counties and even communities, environmental capacity and the nature of the spread of novel coronavirus. “Low-risk areas” are areas where no new cases are confirmed for 14 consecutive days. “Medium-risk areas” have newly confirmed cases within 14 days, but the cumulative number of confirmed cases does not exceed 50, or the cumulative number of confirmed cases exceeds 50, and no cluster epidemic occurs within 14 days. “High-risk areas” refers to more than 50 cumulative cases and a cluster of epidemics occurred within 14 days. People can enter the “State Council Client” applet and perform the “Inquiry on Epidemic Risk Level” to show which epidemic risk level each person is in. For instance, on February 17, 2021, the data base showed 1 high-risk region and 5 medium-risk regions [34]. People are still required to go outside with a mask, take their temperature, and show a health code and a negative certificate of nucleic acid.

**Table 3:** Prevention and control measures taken by the Chinese government in areas with different levels of risk [12, 32, 33]

Measures	High-risk areas	Medium-risk areas	Low-risk areas
Area traffic control	√		
Close public facilities	√		
Close business	√		
Close primary schools and kindergartens	√		
Close colleges and universities	√		
Delayed start of school	√	√	√
Gathering activities are prohibited	√	√	Reduce crowd gathering activities
Suspected cases are quarantined	√	√	
Close contacts are subject to isolation medical observation	√	√	√
Use a mobile app	√	√	√
Comprehensive screening of fever patients	√	√	Medical institutions strengthen the monitoring, detection and reporting of fever cases
Door to door testing	√	√	
Wear masks in public places	√	√	√
Measuring body temperature	√	√	√
Keep social distance	√	√	√
Information registration of outsiders	√	√	√
Check health code, itinerary card	√	√	√
Negative nucleic acid test certificate	√	√	√
Disinfect relevant places	√	√	√

It has been questioned whether China’s official statistics represent the real situation. Some argue that the number of cases and deaths during the peak of the epidemic must have been much higher than presented in the official statistics [35], while others question that the disease could disappear completely from Wuhan [36]. While these arguments might be true, it is a matter of fact that China managed to bring life in Wuhan back almost to normal within a short period. A second wave with tremendous consequences for the public health situation could not have been hidden. Thus, irrespective of the reliability of the statistics basis, the measures taken by the Chinese government must have been quite successful in containing the outbreak and in preventing a second or third wave of outbreaks. In other words: The measures shown in Table 3 must have made it possible to keep the basic reproductive rate low.

Consequently, we have to analyze the parameters determining the dynamics of the spread of COVID-19 in order to understand how the second wave was prevented in Wuhan. For this purpose, we develop a simple and basic model predicting the spread of the disease in the region in order to analyze the influence of different parameters on it.

## Methods

### Modelling COVID-19 – an overview

An epidemic is terminated if the net reproductive rate (*N*_*t*_) at a point of time t is lower than one, i.e., if every newly infected will infect less than one other person. *N*_*t*_ is the product of the basic reproductive rate (*R*_*0*_, under the condition that nobody is immune) with the likelihood that the contact partner is not immune, i.e., at a given share of immune population (*x*_*t*_) at a point of time t, *N*_*t*_ can be calculated as

$$ {N}_t=\frac{R_0\left(100-{x}_t\right)}{100} $$

If *N*_*t*_ is less than 1, a population has reached herd immunity (e.g. 60% for *R*_*0*_ = 2.5).

Consequently, we have to analyze the dynamics of the diffusion of COVID-19 and estimate *R*_*0*_ in order to assess the factors contributing to the success of interventions against the diseases in Wuhan. A huge variety of mathematical models has been developed to forecast the spread of a disease. The simplest approach calculates the basic reproductive rate as a function of some of variables (analytical models). As early as 1889, this model type was developed to calculate R_0_ for malaria (reprint in English in 1989 [37]) and became the foundation of the well-known Ross-MacDonald model [38]. The disadvantage of these simple models is that they cannot cover interdependencies and changes of variables.

Homogenous Markov models are also widely used to forecast the spread of a disease [39]. They are capable to estimate the number of individuals in different health states as long as the transition probabilities are constant, i.e., if they do not depend on the number of individuals in the compartments [40]. This is the case for chronic-degenerative diseases, but the probability of being infected depends on the infectious population. Thus, traditional (homogeneous) Markov models are not applicable for infectious diseases such as COVID-19.

**Fig. 2:**
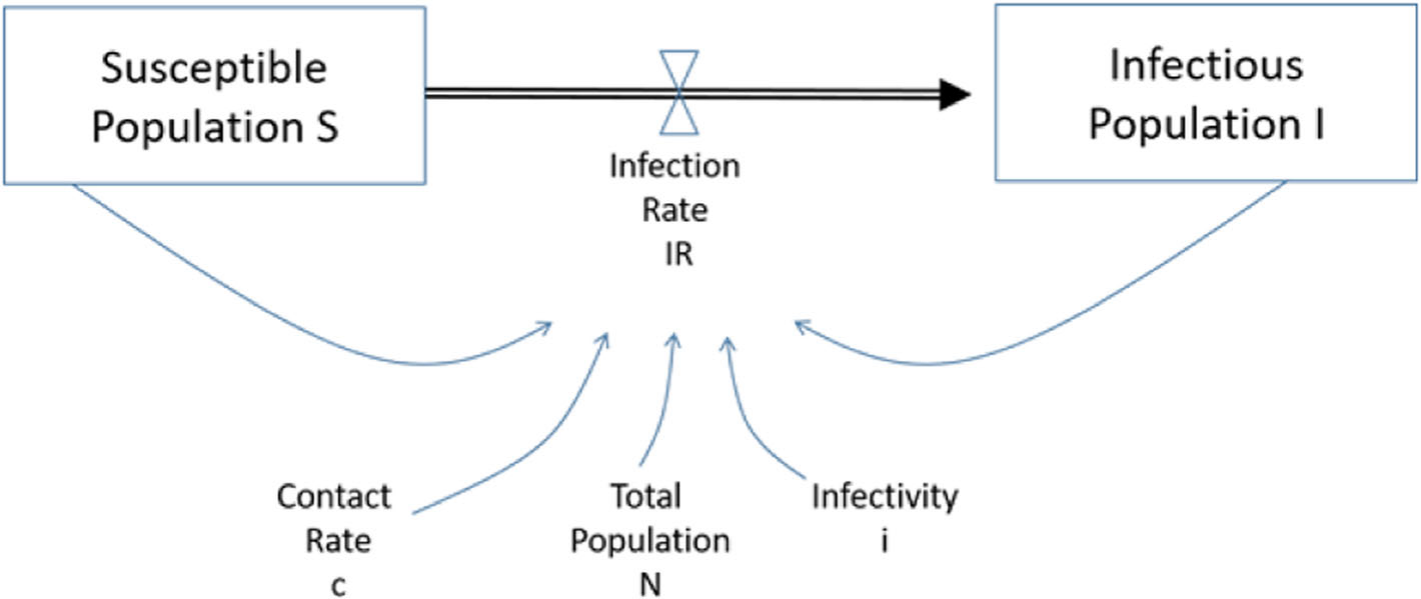
SI-Model. Source: [45]

An inhomogeneous Markov chain implies that the transition probabilities can change. It is, in principle, a system dynamics model. This type of model was developed by Forrester in 1964 [41] in order to account for feed-back loops (e.g. number of infectious population determining the risk of being infected). The principle has been applied in many fields, such as “Industrial Dynamics “[41], “World Dynamics “[42], “Urban Dynamics” [43] or “Disease Dynamics” [44]. The simplest system dynamics model of a disease is the so-called SI-model where S denotes the population susceptible to a disease and I the infectious population. Figure 2 shows that the infection rate depends on the susceptible population (S), the infectious population (I), the contact rate (c), the total population (N) and the infectivity (i) of the infectious disease. The model can be easily enhanced to include the recovered population (SIR-model), exposed population (SEIR-model), different age-sets, re-infections, vaccinations, locations etc. The model has been applied to many infectious diseases, such as HIV/AIDS, malaria and cervical cancer [46–48].

Discrete Event Simulations (DES) and Agent-Based Simulations have also been used to predict the spread of infectious diseases [49, 50]. The advantage of these models is that they do not simulate compartments but individuals allowing to attach personal characteristics (e.g. being a child of an individual mother or having certain comorbidities) to each person. Thus, they are more precise, but require many input variables frequently unknown. In addition, designing and validating these models is much more effort than for the other model types.

In principle, a model should not be more complex than necessary to give an answer to the specific research question. For the target of this paper of determining the reasons for the successful fight against COVID-19 in Wuhan, a rather simple system dynamics model seems appropriate. There is a tremendous number of COVID-19 models available. Stegmaier lists 53 different models of COVID-19 relevant to German public health research, the majority of them system dynamics [51]. However, he also makes aware of the weaknesses of these models, such as poor data input, wrong assumptions, poor transparency, selective reporting etc. Ioannidis, Cripps and Tanner even state that “Forecasting for COVID-19 has failed” [52] because the results were frequently unreliable and of limited value for decision-makers.

The objective of the model presented in the next section is not to forecast the future development of COVID-19 in Wuhan. Instead, we would like to focus on very few parameters influencing the spread of the disease and analyse how they must have developed in order to allow the epidemic dynamics of COVID-19 in Wuhan. While many of the models presented by Stegmaier [51] are much more complex than ours, we also do not pretend to give a precise forecast. Our intention is a “modelling for insights, not for numbers” [53], i.e., we want to learn more about the prerequisites of the real spread of the disease than about the future dynamics.

### Modelling COVID-19 – a basic model for Wuhan

**Fig. 3:**
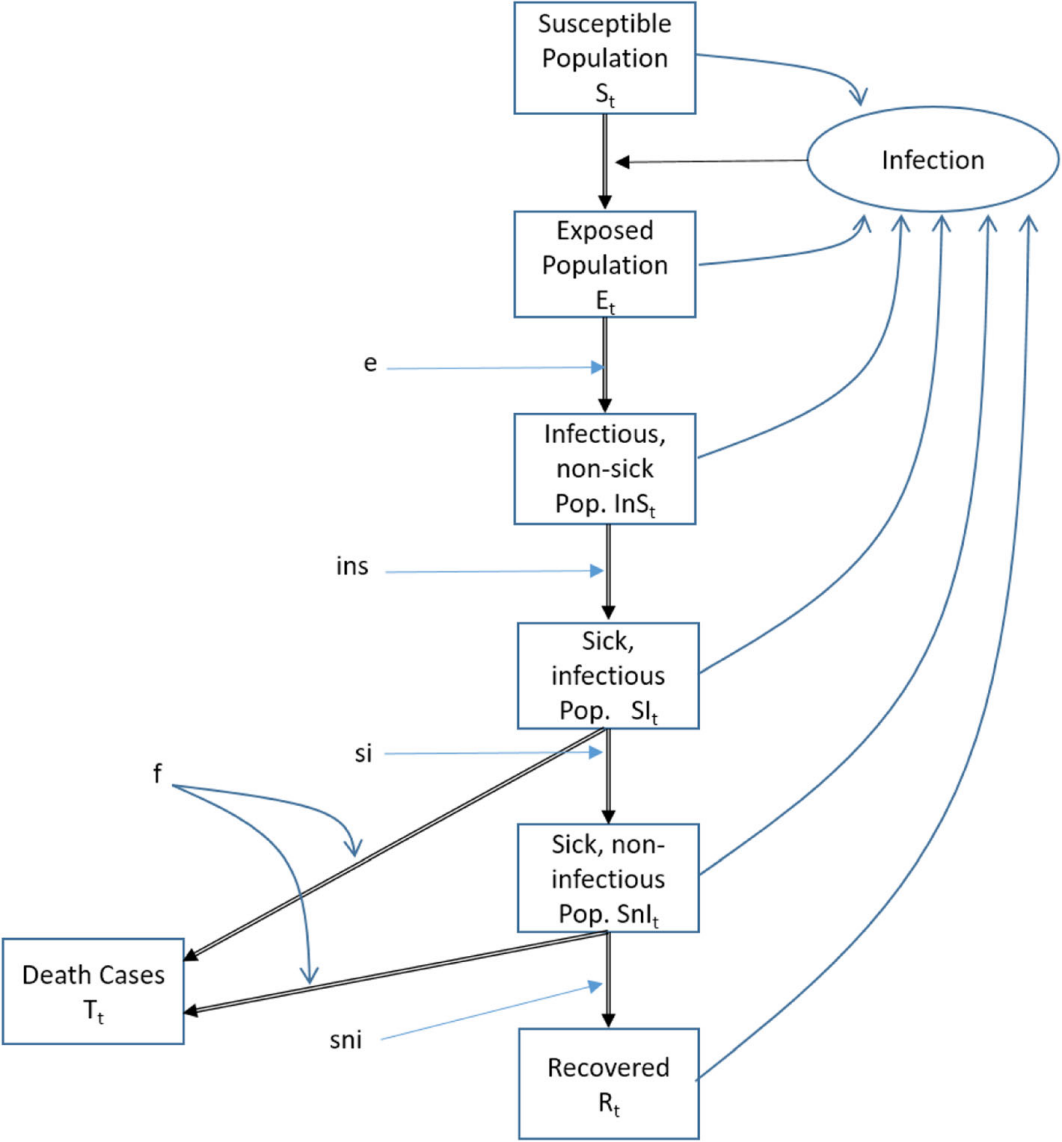
COVID-19 Model Structure. Source: [54]

For this purpose, we develop a generic COVID-19 model [54] in order to analyze the factors determining the spread of the disease in Wuhan in the first year. The model does not consider age-sets, locations or social differentiations (e.g. schools, universities, traditional markets) as this is not necessary to answer the question how China managed to avoid a second wave. Instead, we focus on the determinants of the basic reproductive rate R_0_. The infection life cycle is presented in Fig. 3 and modelled as a System Dynamics Models [55, 56].

The system dynamics model defines difference equations for the healthy, infected, sick and immune population:

$$ {S}_{t+1}={S}_t-\frac{S_t}{S_t+{E}_t+{InS}_t+{SI}_t+{SnI}_t+{R}_t}\cdotp \frac{InS_t+{SI}_t}{S_t+{E}_t+{InS}_t+{SI}_t+{SnI}_t+{R}_t}\cdotp {S}_t\cdotp \frac{R_0}{si+ ins} $$

$$ {E}_{t+1}={E}_t+\frac{S_t}{S_t+{E}_t+{InS}_t+{SI}_t+{SnI}_t+{R}_t}\cdotp \frac{InS_t+{SI}_t}{S_t+{E}_t+{InS}_t+{SI}_t+{SnI}_t+{R}_t}\cdotp {S}_t\cdotp \frac{R_0}{si+ ins}-\frac{E_t}{e} $$

$$ {InS}_{t+1}={InS}_t+\frac{E_t}{e}-\frac{InS_t}{ins} $$

$$ {SI}_{t+1}={SI}_t+\frac{InS_t}{ins}-{SI}_t\cdotp \frac{f}{\left( si+ sni\right)}-\frac{SI_t}{si} $$

$$ {SnI}_{t+1}={SnI}_t+\frac{SI_t}{si}-{SnI}_t\cdotp \frac{f}{\left( si+ sni\right)}-\frac{SnI_t}{sni} $$

$$ {T}_{t+1}={T}_t+\left({SnI}_t+{SI}_t\right)\cdotp \frac{f}{\left( si+ sni\right)} $$

$$ {R}_{t+1}={R}_t+\frac{SnI_t}{sni} $$

$$ {R}_0={\sum}_{i=1}^m\left(1-{\left(1-p\right)}^{n_i}\right) $$

With the following variables and constants:

**Table:** 

Variables	Description
S_t_	Susceptible in t
E_t_	Exposed in t
InS_t_	Infectious but not sick in t
SI_t_	Infectious and sick in t
SnI_t_	Sick and non-infectious in t
R_t_	Recovered in t
T_t_	Death cases in t
R_0_	Basic reproductive rate
N_t_	Net reproductive rate in t

**Table:** 

Constants	Description
f	infection fatality rate
e	Average length of stay in exposed compartment
ins	Average length of stay in compartment infectious but not sick
si	average length of stay in compartment sick and infectious
sni	average length of stay in compartment sick not infectious
$\overline{R_0}$	Basic reproductive rate without intervention
$\overline{R_i}$	Basic reproductive rate with intervention
d_1_	last day without intervention
d_2_	first day of maximum effect of intervention
d_3_	last day of intervention
d_4_	last day of effect of intervention
p	infectivity
n_i_	number of contacts with person i during infectious period
m	number of persons met during infectious period

In comparison to other models [51], the infection life cycle and the number of compartments is rather simple, but we focus much more on the impact of contact rates on the basic reproductive rate. As (8) shows, R_0_ depends on the infectivity (i.e. probability that one contact of an infectious person with a healthy person leads to an infection), the number of people an infectious person meets within the infectious period and number of contacts the infectious person has with each of the healthy persons.

The probability that an infectious person infects a healthy person when meeting once is *p*. The probability that an infectious person does not infect a healthy person when meeting this person *n*_*1*_ times is ${\left(1-p\right)}^{n_1}$. Thus, if an infectious person meets *m* healthy people during the infectious period and has *n*_*i*_ contacts with each of them during this time, is the basic reproductive rate and can be calculated [57] as

$$ \left(8\mathrm{a}\right)\ {R}_0=\left(1-{\left(1-p\right)}^{n_1}\right)+\left(1-{\left(1-p\right)}^{n_2}\right)+\dots +\left(1-{\left(1-p\right)}^{n_m}\right)={\sum}_{i=1}^m\left(1-{\left(1-p\right)}^{n_i}\right) $$

**Fig. 4:**
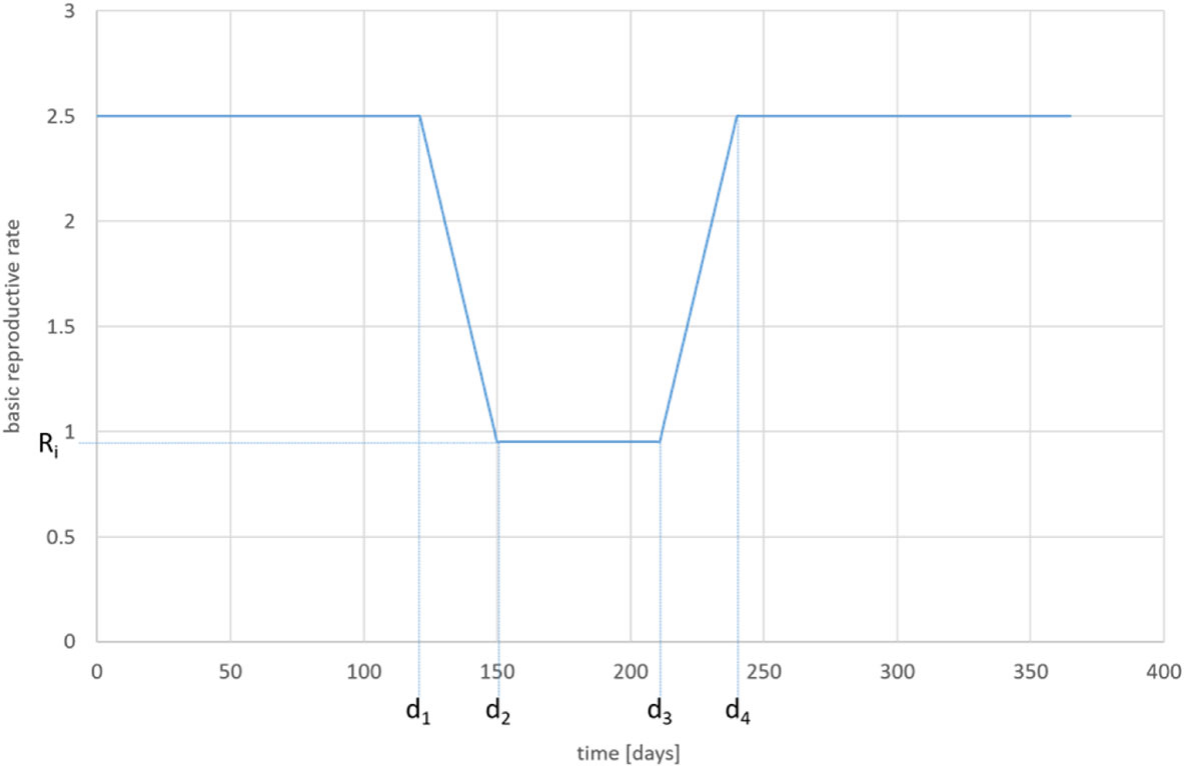
Development of R_0_ (assumption). Source: own

For our analysis of the COVID-19 diffusion in Wuhan, we assume that the disease spread without interventions for a certain time. After *d*_*1*_, the public health care system started interventions resulting in a reduction of *R*_*0*_. However, it took some time until the rate had declined strongly. At *d*_*2*_ all measures reached their maximum effectiveness and this condition was sustained until *d*_*3*_. Afterwards the interventions were relaxed until the old situation was reached again in *d*_*4*_. The respective development is presented in Fig. 4. This can be presented in the formula:

$$ (9){R}_0=\left\{\begin{array}{cc}\overline{R_0}& for\ t\le {d}_1\\ {}\overline{R_0}-\frac{\overline{R_0}-\overline{R_i}}{d_2-{d}_1}\cdotp \left(t-{d}_1\right)& for\ {d}_1<t<{d}_2\\ {}\overline{R_i}& for\ {d}_2\le t\le {d}_3\\ {}\overline{R_i}+\frac{\overline{R_0}-\overline{R_i}}{d_4-{d}_3}\cdotp \left(t-{d}_3\right)& for\ {d}_3<t\le {d}_4\\ {}\overline{R_0}& for\ t>{d}_4\end{array}\right. $$

Consequently, the net reproductive rate (*N*_*t*_) can be calculated as

$$ {N}_t=\frac{R_0\cdotp {R}_t}{S_t+{E}_t+{InS}_t+{SI}_t+{SnI}_t+{R}_t} $$

Based on this model, we can simulate the diffusion of COVID-19 in a generic region with many characteristics of Wuhan. We simulate under the assumption that no intervention had been taken (scenario I), that the reduction of the basic reproductive rate was sustained (scenario II) and that a successful intervention was relaxed too early so that basic reproductive rate returns to its original value (scenario III). The last scenario assumes that some measures are sustained but *R*_*0*_ will be above 1 (scenario IV). Furthermore, we can model the impact of different rates of infectivity (*p*), number of different contact partners (*m*) and contacts per partner (*n*_*i*_). Under the assumption that an infectious person has the same number of contacts with each person (for *n*_*1*_ *= n*_*2*_ *= … = n*_*m*_ *= n*), we receive

$$ \left(8\mathrm{b}\right)\ {R}_0={\sum}_{i=1}^m\left(1-{\left(1-p\right)}^n\right)=m\cdotp \left(1-{\left(1-p\right)}^n\right) $$

or

$$ \left(8\mathrm{c}\right)\ n=\frac{\ln \left(1-\frac{R_0}{m}\right)}{\mathit{\ln}\left(1-p\right)} $$

$$ \left(8\mathrm{d}\right)\ m=\frac{R_0}{1-{\left(1-p\right)}^n} $$

Finally, we can calculate the basic reproductive rate under the assumption that the total number of contacts as the product of people met and contacts per person is constant (for m*n = k = const) as

$$ \left(8\mathrm{e}\right)\ {R}_0=m\cdotp \left(1-{\left(1-p\right)}^{\frac{k}{m}}\right) $$

## Results

For our simulation, we used data from Wuhan without assuming that the model will present all dimensions of the reality of this region. Table 4 shows the parameters. In some cases we could not build on the standard parameters used in other models because we wanted to simulate the situation in the very beginning of the pandemic where very little was known about the disease. For instance, the fatality rate in Wuhan was most likely higher than it is reported for other locations today because hardly anything was known about the diagnostics and therapy of the disease. For these parameters, we built on assumptions and private communication from Chinese experts.

**Table 4:** Basic parameters

Constants	Description	Value	Source
*S*_*0*_	Population in t = 0	11,000,000	
f	infection fatality rate	0.015	[58]+ p.i.^a^
e	Average length of stay in exposed compartment	3	[59] + p.i.^a^
ins	Average length of stay in compartment infectious but not sick	2	[59] + p.i.^a^
si	average length of stay in compartment sick and infectious	11	[59] + p.i.^a^
sni	average length of stay in compartment sick not infectious	7	[59] + p.i.^a^
$\overline{R_0}$	Basic reproductive rate without intervention	2.5	[60]
$\overline{R_i}$	Basic reproductive rate with intervention	Scenario I: 2.5 Scenario II: 0.95 Scenario III: 2.5–0.95-2.5 Scenario IV: 2.5–0.95-1.5	assumption
*d*_*1*_	last day without intervention	120	assumption
*d*_*2*_	first day of maximum effect of intervention	150	assumption
*d*_*3*_	last day of intervention	210	assumption
*d*_*4*_	last day of effect of intervention	240	assumption
*p*	infectivity	0.1	[61, 62]

^a^pi: assumption based on private communication with Chinese colleagues

**Fig. 5:**
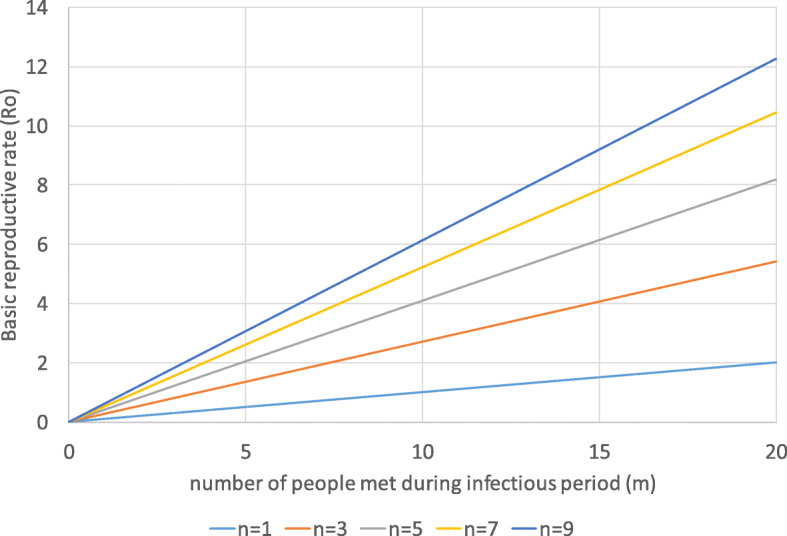
Basic reproductive rate and contacts. Source: own

The original basic reproductive rate is assumed as 2.5 ($\overline{R_0}$) [60]. According to (8d), this refers to 10.8 contact partners with an average frequency of 2.5 meetings per contact during the infectious period and an infectivity of 0.1 (*p*) [61, 62]. Figure 5 shows the relationship between the number of contact persons (*m*), the number of contacts per contact person (*n*) and the basic reproductive rate for *p* = 0.1. It is obvious that both variables strongly determine *R*_*0*_.

**Fig. 6:**
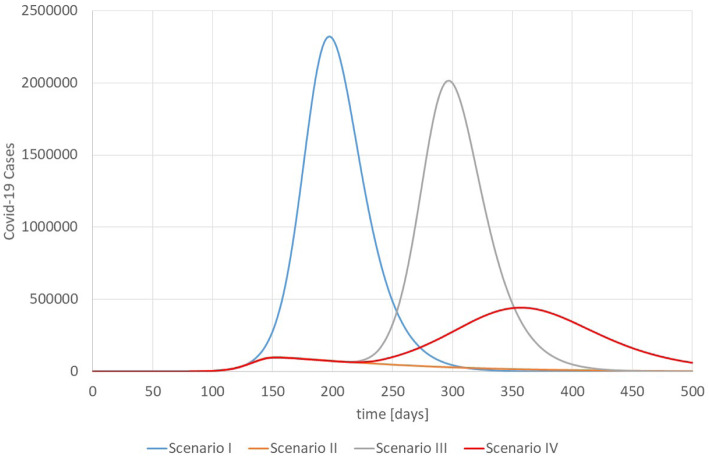
Number of COVID-19 Cases in Wuhan, Scenarios I-IV. Source: own

Figure 6 shows the number of COVID-19 cases for the scenarios. Scenario I assumes that R_0_ = 2.5 is constant, i.e., without any intervention. Scenario II assumes that interventions start at day 121 (d_1_ = 120) and need 30 days (d_2_ = 150) until they are fully effective so that $\overline{R_i}$ =0.95. Afterwards all interventions are sustained. This parameter was not chosen because we have evidence that the reproductive rate was exactly 0.95 in Wuhan. Instead, it is an assumption of a reproductive rate lower than but close to 1.

Scenario III assumes the same development as scenario II for the first 210 days, i.e., the interventions are sustained to 60 more days. Afterwards (d_3_ = 210) the interventions are relaxed until $\overline{R_i}$ is back to its original value of 2.5 in d_4_ = 240. Scenario IV follows the pattern of scenario III but assumes that some interventions are sustained so that the final $\overline{R_i}$ is 1.5.

For the unrealistic case of no interventions (scenario I), Wuhan would have experienced a very severe single wave. Most striking, it takes 87 days after the first case until 1000 patients are sick (variables *SI* and *SnI*) at the same time (10 per 100,000 inhabitants in Wuhan), i.e., the early break-out of the disease is difficult to detect even though the disease has a catastrophic potential leading to thousands of new cases per day. Scenario I is unrealistic as the health care system would have collapsed completely without interventions. The epidemic comes to a standstill after the herd immunity of 60% is reached. Scenario II shows that the interventions are effective and manage to flatten the curve. However, as no herd-immunity is reached, COVID-19 will not disappear and there remains a need to sustain the interventions indefinitely (without vaccination). According to (8d), the number of contact persons must be less than 4.1 if *p* = 0.1 and *n* = 2.5 in order to achieve an *R*_*i*_ = 0.95.

Scenario III simulates the second wave of the COVID-19 pandemic for Wuhan under the assumption that interventions are relaxed completely on day 210. The consequences are disastrous: An unrestricted second wave is much more dramatic than the first wave for scenario II and almost as strong as the first wave without any interventions (scenario I).

Scenario IV assumes – like scenario III – that the interventions are reduced after a period of successful reduction of infections, but some measures are sustained so that R_i_ returns to 1.5 on d_4_ = 240. The consequence is a “milder” second wave, which is still stronger than the first wave but not as dramatic as the second wave of scenario III.

Consequently, the basic reproductive rate must be kept below 1 for a very long time. Based on (8) this can be done by reducing the infectivity (*p*), number of contact partners (*m*) and number of contacts per partner (*n*).

Thus, at a rate of *R*_*0*_ = 2.5, herd immunity is reached if 60% of the population have been infected. At a rate of *R*_*0*_ = 1.5, the respective figure can be 33.3% under the assumption that the number of contacts remains on this low level.

**Fig. 7:**
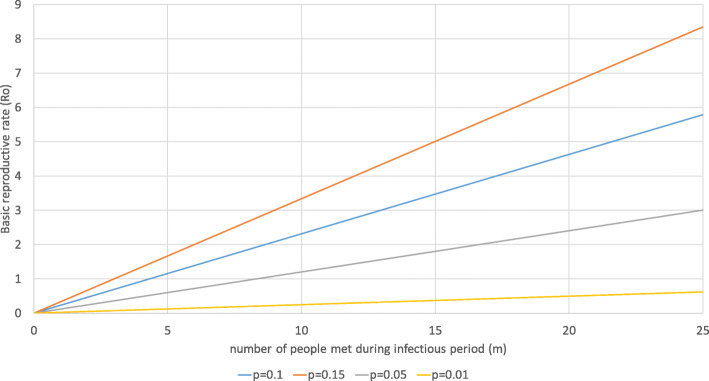
Change of infectivity. Source: own

Figure 7 shows the consequences of a changed infectivity (*p*) on the basic reproductive rate. If *p* increases from 0.1 to 0.15 (as for “UK variant” B.1.1.7), *R*_*0*_ strongly increases. Assuming that a person meets any other person 2.5 times on averages, the increase of *p* by 50% requires that the number of people met during the infectious period declines from 10.8 to 7.5 (see 6d). At the same time, a reduction of the infectivity by wearing surgical masks (estimated effectiveness of 50%) for all contacts allows to increase the number of contact partners to 20.8 for the same *R*_*0*_. Wearing an FFP-2 mask (estimated effectiveness of 90%) for all contacts has a very strong impact on the basic reproductive rate. An infected person can meet 40.3 different people on average 2.5 times during the infectious period and still *R*_*0*_ is below 1 if all contacts are with an FFP-2 mask.

Based on (8d), we can calculate that a *R*_*0*_ of 2.5 will result if an infectious person meets 11 different people on average 2.5 times during the infectious period (*p* = 0.1). By wearing a surgical mask with an effectiveness of 50% (*p* = 0.05), R_0_ will decline to 1.32, i.e., scenario IV can be implemented by merely sustaining the obligation of wearing surgical masks for all contacts. Scenario II could be achieved by wearing surgical masks and by reducing the number of contacts with different people from 10.8 to 7.5 during the infectious period with an average number of meetings per person of 2.5 (*R*_*0*_ = 0.96). Thus, the system is highly sensitive to changes of the infectivity *p*, i.e., wearing effective masks for all contacts is one of the most efficient interventions.

**Fig. 8:**
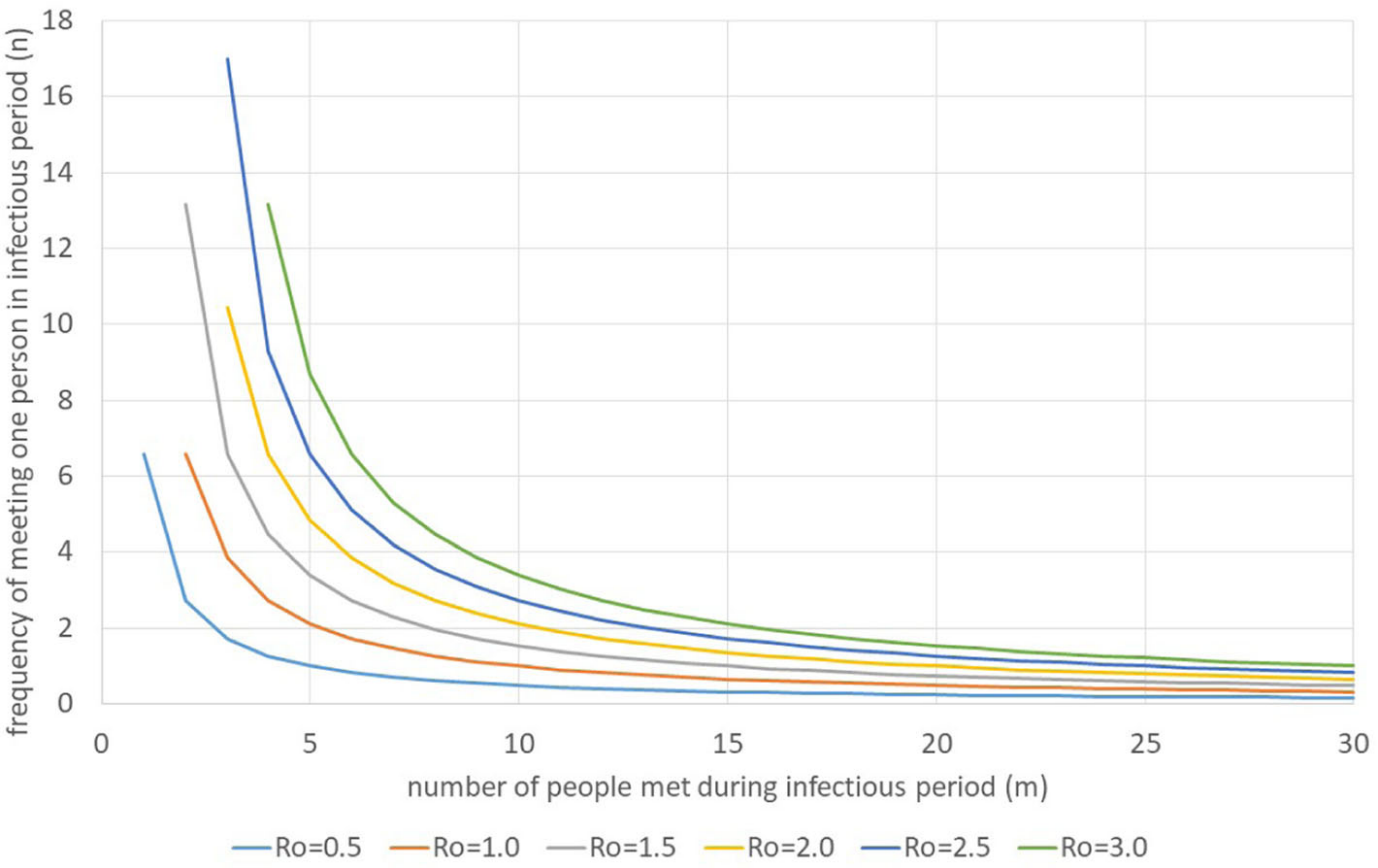
Impact of number of people met and frequency of meeting each person during infectious period. Source: own

Figure 8 shows the impact of different numbers of contacts and different frequencies of meeting each person under the assumption *n*_*1*_ *= n*_*2*_ *= … = n*_*m*_ *= n.*

**Fig. 9:**
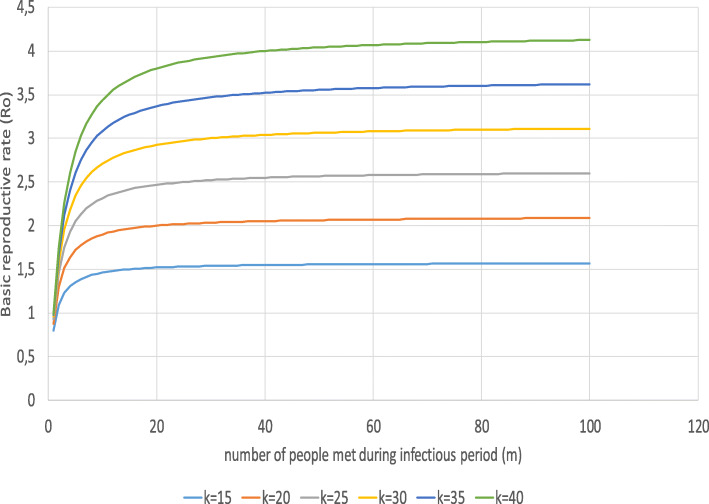
Basic reproductive rate for different total contacts. Source: own

For instance, if an infectious person meets 20 different people during the infectious period, he can meet each of them on average 1.27 times during the infectious period in order to achieve a basic reproductive rate of 2.5. For an *R*_*0*_ of 1, the average number of contacts must decline to 0.47 at 20 different contacts. Alternatively, the person could meet only two different people, but each one 6.68 time. Figure 9 assumes that the total number of contacts is given and the number of people met during the infectious period varies. It is obvious that it is better to meet few people frequently than many people rarely.

## Discussion

### Relevance of simulation results

Based on these calculations we can state that the public health care system in Wuhan managed to reduce the risky contacts strongly. The success of keeping *R*_*0*_ under 1 for several months can only be explained by effective efforts to exclude infectious people from contacts.

The results also indicate that it was very difficult in the beginning of the epidemic to see its pandemic potential. Our simulations show that it took almost 3 months after the first case until 1000 patients were sick at the same time. It is obvious that the traditional routines of case detection (focusing on the number of cases) could not work with COVID-19. Once the figures are visible, it is already too late and the exponential growth has started. Only an excellent public health system could have determined the pandemic potential early enough.

Our results also indicate that it is likely that Wuhan had much more cases and deaths in the first wave than reported. Even scenario II results in 8269 death cases in the first year while Wuhan reported “only” 2997. The statistics of Wuhan have been questioned elsewhere [63, 64] and our computations show equal results.

The simulations also show that wearing surgical masks is highly effective to reduce the basic reproductive rate and the spread of the epidemic. Our simulation results are highly sensitive to changes of the infectivity, which can be strongly influenced by wearing masks. This instrument of protecting oneself and others has been quite common in China before, but it has become almost a universal habit since the pandemic started. Chinese citizens wear surgical masks, not only in public transport but almost everywhere. It has become a common habit as a population response to air pollution [65] and hardly anybody would see it as an insult to their liberty rights as citizens.

Finally, the simulation results show that a second wave can only be avoided if interventions are sustained. The reduction of the medical infectivity (*p*), the number of contact persons (*m*) and the number of contacts per contact person (_n_) is the key to control the pandemic. It seems that China managed well to sustain a low *R*_*0*_ by controlling these variables.

### Geography

There was some discussion in the beginning whether Wuhan managed to control the pandemic because of the geographical location and the respective climate [66]. However, while other states located at the same altitude (e.G. *Florida*, Louisiana, Texas, Egypt) are facing a second wave, Wuhan has not reported Corona cases since March, i.e., the geographical location cannot explain the difference. Without doubt, spring and summer helped to control COVID-19 in Wuhan in 2020. There is a clear negative correlation between temperature and COVID-19 incidence, but for other parameters (e.g. humidity, wind speed, rain fall) the results are not significant [66]. It is likely that temperature does not have a direct impact on the transmission of the virus but increases the parameter *m* and *n*, i.e., during the cold season people have more and closer contact in rooms. However, this argument is true for all cities on the same latitude and does not explain the successful avoidance of a second wave in Wuhan. Geography does not explain this success.

### Public health system

The simulation results also show that an early detection of cases and the implementation of early and effective control measures would require an excellent public health system. However, this does not seem to be the case. Instead, a number of shortcomings of China’s CDC have become visible during the epidemic [67]. Firstly, the communication between the national the local CDCs as well as with the healthcare facilities did not work well. Although an infectious disease information system had been developed after SARS-1, it did not work properly during phase I of COVID-19. Secondly, the CDC of China had a very limited influence on the Government. As early as January 6, 2020, the Chinese CDC initiated the second-level response to the epidemic, which was upgraded to a first-level response on January 15. However, these emergency responses were almost ignored by the Government [1].

**Fig. 10:**
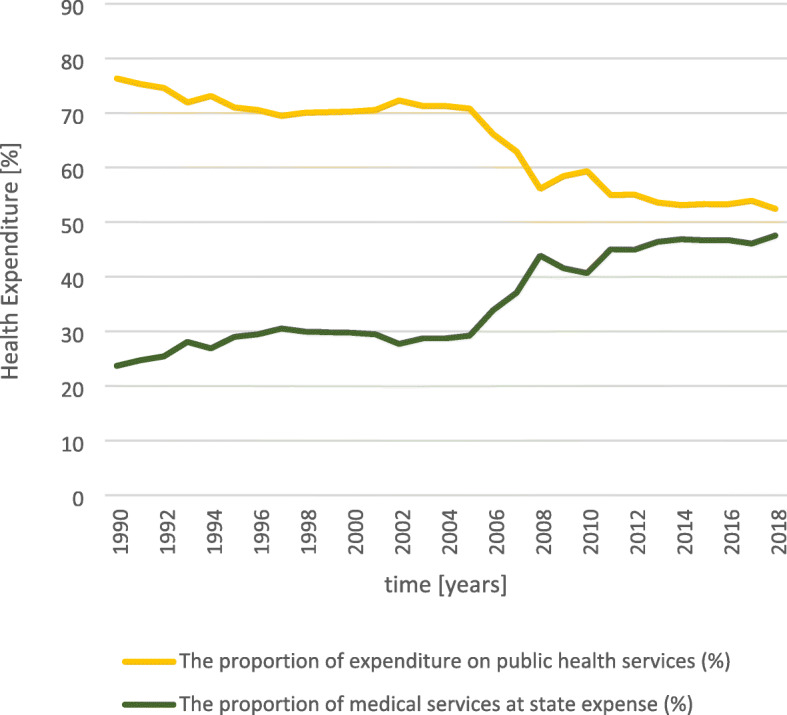
Government expenditure on public health. Source: [70]

Thirdly, the public health system of the middle-income country China suffers from low resources. As shown in Table 5, financial (health expenditure p.c.) and personnel resources of the system are much lower than in high income countries. In particular, the funds allocated to primary services have been declining for years (Fig. 10). The absolute amount of public health expenditure in China increased tremendously from 14.3 billion yuan (2.99 billion US$ or 2.63 US$ p.c.) in 1990 to 860 billion yuan (130 billion US$ or 617 US$ p.c.) in 2018. However, the proportion of preventive and promotive public services in the total public health expenditure decreased from 76.3% in 1990 to 52.5% in 2018. It seems that the Government of China puts less emphasis on prevention than treatment.

**Table 5:** Health resources per capita in China and some high-income countries in the world in 2016. Source: [68, 69]

Country	Current Health Expenditure p.c. [PPP US$]	Hospitals per million population	Number of beds per 1000 population	Doctors per 1000 population	Nurses per 1000 population
Germany	5568.27	37.64	6.06	4.19	10.84
United Kingdom	4182.18	29.29	2.57	2.78	6.45
United States of America	9941.35	17.14	2.77	2.59	–
Japan	4424.98	66.51	13.11	2.43	11.34
Republic of Korea	2745.07	73.92	11.98	2.29	6.82
Canada	4809.28	19.99	2.60	2.69	9.96
Australia	4634.65	55.93	3.84	3.58	9.55
*China*	*762.98*	*21.07*	*4.02*	*1.88*	*2.54*

During the COVID-19 outbreak, the Chinese government has borne the cost of all confirmed and suspected COVID-19 patients. It is estimated that the respective costs amounted to 15.696 billion US$, mainly on treatment of patients, subsidies for epidemic prevention and control personnel, and purchase of equipment and protective materials. In comparison to the total health expenditure, the cost of the epidemic amounted to 1.65% of total public health expenditure (11.21 US$ per capita resp. 0.1% of GNP p.c.), i.e., a rather small amount. Consequently, neither a brilliant, well-financed and well-staffed public health system nor tremendously high health care expenditure are the key to understand the effective control of the Wuhan epidemic.

### Portfolio of interventions

A number of analyses have been published that provide a taxonomy of different interventions against the diffusion of COVID-19 and assess their effectiveness. For instance, Baker et al. [71] listed the components of pandemic control of COVID-19. A comparison with the interventions of Table 3 shows that China has not implemented any measures that are not practiced elsewhere as well. Improvement of hygiene (e.g. hand washing, surgical masks), contact tracing, quarantine of sick and suspected, high volume testing, physical distancing, movement restrictions, and border management (incl. Exclusion and quarantine) are the international standards to fight COVID-19 [71].

Other studies analyzed the effectiveness of interventions in 40 countries. They record the strongest reduction of *R*_*0*_ if gatherings of more than 5 people are banned followed by closing stores, restaurants, bars and schools [61, 72–74]. China implemented all of these intervention measures – so as many other countries that experienced a second wave. Consequently, it seems that there is no “magic bullet” against the pandemic; China has not implemented different measures, but it seems that the timing and intensiveness was different.

China follows a “zero-COVID” strategy. For instance, a recent breakout in Shijiazhuang (10.9 Mio. inhabitants) in Hebei province exemplifies this “no-tolerance against COVID-19”. After the public health system recorded 300 cases (i.e. 2.75 cases per 100,000), the full program shown in Table 3 started. The objective is clearly described by „zero-COVID “[75]. While European countries discuss whether interventions should be relaxed at a rate of 50:100,000, China implements its full portfolio at a rate of 2.75:100,000.

Without doubt, this is only possible with strong limitations of citizen rights. In particular, the Chinese intervention system builds on the App-based location analyses (see section 2.2). Every contact is recorded and access to gatherings is only permitted if the smart phone gives green light. This seems quite restrictive for Western societies. However, China is not alone in its “Zero-COVID” paradigm [76]. For instance, Australia [77], New Zealand [71] and Southern Korea [78] were quite successful in their eradication campaigns. New Zealand, for instance, never wanted to live with COVID-19, but eradicate it. When it started its campaign on March 23, 2020, the country just had about 100 COVID-19 cases and no deaths. As Philippe and Marques have shown for 11 G10 counties, countries following this strategy of early elimination are epidemiologically and economically more successful that countries pursuing a mitigation or suppression strategy [78]. This “go early go hard’ approach is exactly what China is doing – it seems to work even in a liberal Western society like New Zealand [79].

Finally, China invests efforts to vaccinate its population against SARS-Cov-2 [80]. However, there is evidence that the combination of limited coverage (i.e. share of population able and willing to be vaccinated) and effectiveness of the vaccine will now allow to reduce completely the other interventions [81]. A certain part of the population will not be vaccinated because they will refuse or because age and/or health conditions do not allow [82, 83]. Moreover, the effectiveness of the vaccine to prevent the spread of the disease might be less than 90%. Consequently, there will be (smaller) waves of COVID-19 after the vaccination program will have been completed. Consequently, the instruments described in Table 3 will still have to be employed for a longer time.

### Limitations

The results presented in this paper are subject to a number of limitations. Firstly, we did not model and simulate the precise reality of Wuhan. For a detailed analysis we would have to distinguish age-sets, locations (e.g. city quarters) and social interaction (e.g. schools, work place etc.). Our model is generic, but it permits the conclusion that the public health care system of China managed to control the most important parameters (number of persons contacted and number of contacts per person).

Secondly, some of the data applied to the simulation are uncertain. For instance, as the real number of infections in Wuhan is unknown (and will most likely remain unknown for political reasons) it is difficult to assess the infection fatality rate (f). As Meyerowitz-Katz & Merone show [58], the parameter f strongly differs from place to place with an average of 0.68% and a highest estimate of 1.7%. We assume that the case and consequently the infection fatality rate was towards the higher end in Wuhan in January and February 2020 as no diagnostic and treatment standards had been developed for COVID-19 patients. However, we are aware of the fact that this is an assumption.

For scenario II, an f of 0.015 (see Table 4) results in 8269 death cases within the first year, an f of 0.02 in 10,745, an f of 0.01 in 5656 and an f of 0.005 in 2901 death cases. Consequently, the results react on changes on the parameters, but it is difficult to believe that medical care in Wuhan in the first months of the unknown diseases was as effective as health care systems that had months to learn how to diagnose and treat COVID-19 patients. Therefore, the simulation results might be challenged because of the uncertainty of input data, but the general finding that the number of death cases must be higher than reported is still valid.

Finally, the model presented in this paper does only present the situation in Wuhan in the first year of the epidemic. Consequently, we did not consider vaccination programs, temporary immunity or re-infections. As our objective was the analysis of the public health response in Wuhan in 2020, there was no need to include these aspects. Further research will have to focus on these issues much more.

Summarizing we can state that the model presented in this paper must not be used to predict the future spread of the disease. Instead, it is “modelling for insights, not for numbers” [53].

## Conclusions

Although daily life in Chinese schools and work places is almost back to normal, China has maintained a number of interventions against COVID-19 until today (as of February 2021). Surgical masks and social distancing are a must in all public places, travelling abroad and visiting friends is strongly restricted, and access to public gatherings is only permitted if the smartphone app shows “green”. The app “Health Code” has become the daily companion of all citizens.

As our simulations demonstrate, a return to “normal” life with the same frequency and intensity of contacts as before the intervention would inevitably trigger a second wave if sufficient herd immunity had not previously been achieved. Assuming an *R*_*0*_ of 2.5 for COVID-19, the herd immunity would have to be around 60%, i.e., 60% of the population would have to be immune against the virus to eradicate the disease. Even assuming that 90% of the infections in Wuhan were asymptomatic [64], the herd immunity would be about 40%, i.e., Wuhan is still at risk of COVID-19. Apparently, with the measures taken it is possible to keep the effective reproduction number below 1.

China has not implemented unique interventions. Masks, social distancing and mass testing are well-known instruments all over the world. The “secret” of China’s success in fighting COVID-19 seems to be the early reaction and rigor with which the public health system reacts at comparably low prevalence rates. Currently, the epidemic situation shows a pattern of sporadic and concentrated outbreaks in local areas. Until the herd immunity is reached by the (ongoing) vaccination program, the interventions will have to be maintained. Local outbreaks of COVID-19 were in urban areas with strict control of the population. Whether a rural outbreak could be managed as effectively in China, is questioned [84].

From a European perspective, door-to-door inspections and tight controls via apps are seen as serious violations of individual rights. However, these measures have prevented a second wave and saved lives so far. Some other countries have started seeing the mobile location data technology as an important component in the fight against COVID-19 without sacrificing citizen rights, such as the General Data Protection Regulation (GDPR) of the EU [85, 86]. Learning from the successful intervention program of China does not mean copying the entire portfolio of instruments, but it requires reflecting on the pros and cons of the instruments.

## Data Availability

Free

## Abbreviations

CDC: Center for Disease Control
COVID-19: Corona Virus Disease 2019
ICTV: International Committee on The Classification of Viruses
NCIP: Novel Coronavirus Infected Pneumonia
2019-nCoV: 2020-Novel Coronavirus 2019
NHC: National Health Commission
PCR: Polymerase Chain Reaction
USA: United States of America
WHC: Wuhan Health Commission
WHO: World Health Organization
